# Paraoxonase 2 Deficiency Causes Mitochondrial Dysfunction in Retinal Pigment Epithelial Cells and Retinal Degeneration in Mice

**DOI:** 10.3390/antiox12101820

**Published:** 2023-09-30

**Authors:** Parameswaran Gangadharan Sreekumar, Feng Su, Christine Spee, Elise Hong, Ravikiran Komirisetty, Eduardo Araujo, Steven Nusinowitz, Srinivasa T. Reddy, Ram Kannan

**Affiliations:** 1 Doheny Eye Institute, Pasadena, CA 91103, USA; sparameswaran@doheny.org (P.G.S.); cspee@doheny.org (C.S.); ehong@doheny.org (E.H.); 2 Department of Neurology, David Geffen School of Medicine, University of California at Los Angeles, Los Angeles, CA 90095, USA; fsu@mednet.ucla.edu; 3 Department of Medicine, Division of Cardiology, David Geffen School of Medicine, University of California at Los Angeles, Los Angeles, CA 90095, USA; rkomirisetty@mednet.ucla.edu; 4 Jules Stein Eye Institute, David Geffen School of Medicine, University of California at Los Angeles, Los Angeles, CA 90095, USA; araujo@jsei.ucla.edu (E.A.); snusinowitz@mednet.ucla.edu (S.N.); 5 Department of Molecular and Medical Pharmacology, David Geffen School of Medicine, University of California at Los Angeles, Los Angeles, CA 90095, USA

**Keywords:** oxidative stress, paraoxonase, sodium iodate, mitochondrial bioenergetics, RPE, retinal function

## Abstract

Although AMD is a complex disease, oxidative stress is a crucial contributor to its development, especially in view of the higher oxygen demand of the retina. Paraoxonase 2 (PON2) is a ubiquitously and constitutively expressed antioxidant protein that is found intracellularly associated with mitochondrial membranes and modulates mitochondrial ROS production and function. The contribution of PON2 to AMD has not been studied to date. In this study, we examined the role of PON2 in AMD utilizing both in vitro and in vivo models of AMD with emphasis on mitochondrial function. Mitochondrial localization and regulation of PON2 following oxidative stress were determined in human primary cultured retinal pigment epithelium (hRPE) cells. PON2 was knocked down in RPE cells using siRNA and mitochondrial bioenergetics were measured. To investigate the function of PON2 in the retina, WT and PON2-deficient mice were administered NaIO_3_ (20 mg/kg) intravenously; fundus imaging, optical coherence tomography (OCT), electroretinography (ERG) were conducted; and retinal thickness and cell death were measured and quantified. In hRPE, mitochondrial localization of PON2 increased markedly with stress. Moreover, a time-dependent regulation of PON2 was observed following oxidative stress, with an initial significant increase in expression followed by a significant decrease. Mitochondrial bioenergetic parameters (basal respiration, ATP production, spare respiratory capacity, and maximal respiration) showed a significant decrease with oxidative stress, which was further exacerbated in the absence of PON2. NaIO_3_ treatment caused significant retinal degeneration, retinal thinning, and reduced rod and cone function in PON2-deficient mice when compared to WT mice. The apoptotic cells and active caspase 3 significantly increased in PON2-deficient mice treated with NaIO_3,_ when compared to WT mice. Our investigation demonstrates that deficiency of PON2 results in RPE mitochondrial dysfunction and a decline in retinal function. These findings imply that PON2 may have a beneficial role in retinal pathophysiology and is worthy of further investigation.

## 1. Introduction

Age-related macular degeneration (AMD) is a complex, progressive retinal degenerative disease that continues to be the leading cause of blindness in elderly populations [1] AMD is estimated to affect 196 million persons globally between the ages of 45 and 85 in 2020, rising to 288 million by 2040 [2]. Between the two forms of AMD, loss of retinal pigment epithelial (RPE) cells and photoreceptors is a hallmark of the more common “atrophic” AMD, and there are no proven treatments to stop RPE cell death or replace RPE cells lost to AMD. Located between the retina and choroid in the posterior part of the eye, the RPE is a pigmented monolayer that holds multiple critical functions such as phagocytosis of the photoreceptor outer segment, recycling of vitamin A, and preserving the blood–retinal barrier [3,4]. 

Paraoxonase 1, 2, and 3 (PON1, PON2, and PON3) are three members that make up the mammalian paraoxonase gene family. In humans, the genes for all three of the proteins are found in the long arm of the seventh chromosome [5]. PON1 and PON3 are mostly produced in the liver and released into the circulation [6]. In contrast to its paralogs, PON2 is almost ubiquitously expressed in all tissues and is found in the endoplasmic reticulum (ER), lysosomes, mitochondria, and perinuclear space and is associated with the plasma membrane [7,8,9,10]. Although PON proteins are very similar in their amino acid sequences, based on their localization patterns they appear to associate with different functions [11].

Accumulating evidence in multiple tissues demonstrated the functional role of PON2 as an antioxidative, anti-apoptotic, and anti-inflammatory molecule. PON2 has been found to have antioxidant properties that can protect against oxidative and ER stress in studies using cell culture and animal models [7,10,12,13,14,15]. PON2 knockdown significantly increased ROS levels, while its overexpression significantly decreased ROS, prevented apoptosis and inhibited cell-mediated low-density lipoprotein oxidation [16]. It has been reported that PON2 protects the human umbilical vein endothelial cells against GA and CML-induced oxidative stress, ER stress, and inflammation [13,14]. PON2 deficiency causes mitochondrial dysfunction in macrophages [16]. Despite a few studies demonstrating altered PON1 activity in eye diseases, including AMD [17], diabetic retinopathy [18,19], and cataracts [18], information on the function and regulation of PON2 in AMD ocular pathophysiology is limited. The participation of RPE in PON2 metabolism and effect is not well understood. In particular, the extent to which mitochondrial function in primary RPE cells is impacted by PON2 deficiency and retinal function in PON2-deficient mice is not clear. The objective of the current investigation was to examine PON2 expression, regulation, and potential antioxidant effects in primary cultured RPE cells and in a well-established preclinical mouse model of atrophic AMD.

## 2. Materials and Methods

### 2.1. Retinal Pigment Epithelium (RPE) Culture

The Declaration of Helsinki’s guidelines for research involving human subjects were followed in all studies. RPE cells were isolated from human fetal eyes that were purchased from Advanced Bioscience Resources Inc. in Alameda, California, and grown as described [20]. In brief, hRPE cells were cultured in Dulbecco’s modified Eagle medium (DMEM, #15-013-CV, Corning, NY, USA) containing 10% fetal bovine serum (FBS, # 4800-500HI, Laguna Scientific, Laguna Niguel, CA, USA). Upon reaching confluency, cells were subcultured, and passages 2–4 were used in all experiments. Two donors were used for experiments, and donor-to-donor variation had an insignificant effect on the results.

### 2.2. Effect of Oxidative Stress on PON2 Expression and Subcellular Localization

RPE cells were grown on 4-chamber slides and received 150 µM tert-Butyl hydroperoxide (tBH; Sigma-Aldrich Corp., St. Louis, MO, USA) treatment for 4 or 24 h on media containing 1% FBS. To visualize the mitochondria, cells were incubated with MitoTracker Red CMXRos (500 nM; # M7512, Waltham, MA, USA) for 10 min in 10% FBS-containing medium before fixation and processing for PON2 staining. After several washings, cells were fixed with 4% paraformaldehyde for 20 min at room temperature, permeabilized with 0.5% TritonX-100 for 5 min, and then blocked for 30 min using Animal-Free Blocker (SP-5035-100, Vector Lab, Newark, CA, USA). Cells were incubated overnight with PON2 antibody (1:50 dilution # SC-373981, Santa Cruz Biotech, Dallas, TX, USA) in a blocking solution. Samples were then washed and incubated in the dark for 30 min with a fluorescence-conjugated secondary antibody (Vector Laboratories in Burlingame, CA, USA). A laser scanning confocal microscope (LSM 710, Zeiss, Thornwood, NY, USA) was used to capture images. Fluorescence intensity was calculated with LSM Software ZEN (Zeiss).

### 2.3. siRNA-Mediated Knockdown of PON2 and Mitochondrial Functional Studies

ARPE-19 cells (passage # 10, ATCC, Manassas, VA, USA) were validated by RT-PCR evidence for the expression of Keratin-18, BEST1, and CRALBP [21]. For all transfection experiments, ARPE-19 cells with a confluence of 40–50% were used. The siRNA targeting human PON2 sequences (Hs-PON2_ FlexiTube siRNA # SI03121027, # SI00006678) (Qiagen, Valencia, CA, USA) and negative control siRNA (Qiagen) were mixed with RNAi MAX transfection reagent (Life Technologies, Carlsbad, CA, USA). Real-time RT-PCR was used to assess PON2 mRNA expression 24 h after transfection. Transfection efficiency was determined by analyzing PON2 mRNA expression by real-time RT-PCR after 24 h post-transfection.

PON2-silenced RPE cells were treated with 300 µM tert-Butyl hydroperoxide (tBH) for 24 h and then examined using the XF96e Extracellular Flux analyzer (Agilent Technologies, Santa Clara, CA, USA) to assess the impact of PON2 on cellular bioenergetic profiles. For each measurement, the cartridges were hydrated overnight according to the manufacturer’s instructions in a non-CO_2_ incubator. The standard growth medium was changed out for 180 µL of XF assay medium (XF DMEM medium pH 7.4 with 5 mM HEPES without phenol red), which contained glucose (25 mM), sodium pyruvate (1 mM), and glutamine (2 mM). The concentration of inhibitors was oligomycin, an inhibitor of complex V, at 1.5 µM; carbonyl cyanide 4-(trifluoromethoxy) phenylhydrazone (FCCP), which depolarizes the mitochondrial membrane, at 0.5 µM; and a combination of rotenone, a compound that inhibits complex I, and antimycin A, a compound that inhibits complex III, at 0.5 µM each. Following analysis, the amount of cellular protein in each well was determined, and the results were used to standardize Seahorse XF Analyzer values.

### 2.4. NaIO_3_-Induced RPE Degeneration Mouse Model

Male mice aged 6 to 8 weeks were used for all experiments. PON2-deficient mice were obtained [22] from the breeding colony of the Department of Laboratory and Animal Medicine at the David Geffen School of Medicine at the University of California, Los Angeles (UCLA). In brief, a mouse embryonic stem cell line (cell line XE661, strain 129/Ola) with an insertional mutation in PON2 was identified using the gene-trapping program BayGenomics [23]. As determined by 5′ rapid amplification of cDNA ends [24], the insertional mutation in XE661 occurred in the second intron of PON2. The mutation results in the production of a fusion transcript consisting of exon 1–2 sequences from PON2 and βgeo. XE661 was injected into C57BL/6 blastocysts to generate chimeric mice on a C57BL/6/129 background. After testing for germ line transmission, chimeric mice were backcrossed an additional ten generations on a C57BL6/J background before intercrossing to obtain wild-type and homozygote knock-out mice for the experiments presented in this manuscript. Male mice (C57BL6/J) were purchased from The Jackson Laboratory (The Jackson Laboratory, Bar Harbor, ME, USA). All animal procedures were approved by the UCLA Institutional Animal Care and Use Committee (# ARC 2019-060). All study procedures were conducted in accordance with National Institutes of Health guidelines and the Association for Research in Vision and Ophthalmology (ARVO) Statement for the Use of Animals in Ophthalmic Vision Research.

The NaIO_3_-induced mouse model of RPE atrophy has been well characterized in our laboratory [25]. Sodium iodate (NaIO_3_) (Sigma-Aldrich Corp., St. Louis, MO, USA) was dissolved in sterile normal saline. Based on our previous studies [25], we used a single dose of 20 mg/kg BW NaIO_3_ administered via the tail vein. The same volume of sterile PBS was injected into the tail vein of the controls. Mice (WT and PON2-deficient) were divided into different groups as outlined below. Group 1: WT + PBS; Group 2: WT + NaIO_3_; Group 3: PON2-deficient + PBS; and Group 4: PON2-deficient + NaIO_3_. Each group consisted of 5-8 mice. All mice were maintained on a standard chow diet. The eyes were enucleated seven days after NaIO_3_ treatment and processed for different staining as described in the respective sections below.

### 2.5. Spectral Domain Optical Coherence Tomography (SD-OCT) and Fundus Imaging

Ketamine/xylazine was injected intraperitoneally to anesthetize mice. Pupils were dilated with one to two drops of Tropicamide Ophthalmic Solution 1% (Bausch & Lomb, Tampa, FL, USA). An ocular demulcent solution comprising hydroxypropyl methylcellulose ophthalmic (Gonak, Akorn, Lake Forest, IL, USA) was administered to the eye to create a consistent, optically transparent interface.

Ultra-high resolution spectral domain optical coherence tomography (SD-OCT) imaging was performed on both eyes at the end of experiments on day 7 (Bioptigen SD-OCT system, Research Triangle Park, Durham, NC, USA) as described [25]. The total retinal thickness—measured as a distance (in µm) between the inner limiting membrane and the retinal pigment epithelium—was determined for each location. The fundus images were acquired with a Micron II retinal imaging microscope (Phoenix Research Laboratories, Inc., Pleasanton, CA, USA).

### 2.6. Electroretinography

Retinal function was evaluated using full-field electroretinograms (ERGs). Following overnight dark adaptation, mice received an intraperitoneal injection of saline containing ketamine (15 mg/kg body weight) and xylazine (7 mg/kg body weight) for anesthesia. ERGs were recorded with the Celeris electrophysiological system (Diagnosys, MA, USA). Pupil dilation was achieved using one drop each of 1% Tropicamide and 2.5% Phenylephrine Hydrochloride Ophthalmic Solutions, and a drop of lubricant eye gel (0.3%, GenTeal Tears) was applied to the corneal surface to ensure good optics and maintain corneal integrity. Body temperature was maintained at 38 °C. Stimuli were delivered to both eyes simultaneously using a fiber-optic bundle with an embedded electrode at each fiber-optic tip. Signal processing was performed using software provided by the instrument manufacturer. Post data collection analyses were performed using custom software. Responses were computer-averaged for each stimulus condition, with up to 50 records for the weakest signals. All stimuli were presented at 1 Hz except for the brightest flashes, where the presentation rate was slowed to 0.2 Hz. Intensity–response functions were analyzed to extract Vmax, which is the maximum saturated b-wave amplitude, for each stimulus condition. Following dark-adapted testing, cone-mediated responses were recorded on a rod-photoreceptor saturating background (32.0 cd/m^2^).

### 2.7. Retinal Histology

Mice were euthanized on day 7 post NaIO_3_ administration. The eyeballs were dissected out, the lens was removed, and the posterior eye cups were fixed in 4% formalin before being embedded in OCT compound and frozen in liquid nitrogen. Cryosections (8 um) were processed for Hematoxylin and eosin (H&E) and sections were scanned on an Aperio digital slide scanner (Leica Biosystems, Buffalo Grove, IL, USA).

### 2.8. Terminal Deoxynucleotide Transferase dUTP Nick End Labeling (TUNEL) Staining

The TUNEL assay was performed on retinal cryosections according to the manufacturer’s (# 12156792910, Roche) instructions. Retinal cryosections (8 µm) were air dried, fixed in 4% paraformaldehyde (PFA), permeabilized with 0.1% Triton X-100 on ice, and followed by incubation with the TUNEL reaction mixture at 37 °C for 1 h in a humid chamber to prevent evaporation. After 3 washes in PBS and mounting, the sites of apoptotic DNA fragmentation were imaged by a digital fluorescence microscope (KEYENCE, Itasca, IL, USA) with a 20× objective. TUNEL-positive cells were counted in five selected areas of each retinal section imaged with a 20× objective. All the cells with a visible nuclear profile that were evident in the visual fields were analyzed and divided into TUNEL-positive and negative. The percentage TUNEL-positive cells is calculated by dividing the averaged TUNEL-positive cells by the averaged total cells in the field of vision.

### 2.9. Immunofluorescence Staining

Following air drying, retinal cryosections were fixed in 4% PFA and permeabilized in 0.1% Triton X-100. Sections were blocked with Animal-Free Blocker for 30 min (Vector Laboratories, Inc., Newark, CA, USA). Sections were incubated with rabbit polyclonal antibody against cleaved caspase-3 (1:100 dilution, #9661, Cell Signaling Tech, Danvers, MA, USA) overnight. Retinal sections were then incubated with fluorescein-conjugated anti-rabbit secondary antibody for 30 min at room temperature. Using a Keyence fluorescence digital microscope, images were acquired. Image J (US NIH, Bethesda, MD, USA) was used to calculate the average total corrected fluorescence (background subtracted) from digital images that were studied (n = 5–6) to quantify immunofluorescence.

### 2.10. Data Analysis

The data were analyzed using a one-way ANOVA followed by a Tukey post-test. PON2 and active caspase fluorescence intensity were compared using unpaired *t*-tests. (GraphPad Prism, version 5; GraphPad Software, Inc., La Jolla, CA, USA). Significant was defined as *p* < 0.05.

## 3. Results

### 3.1. Effect of Oxidative Stress on PON2 Expression in Human RPE Cells, and Its Subcellular Localization

**Figure 1:**
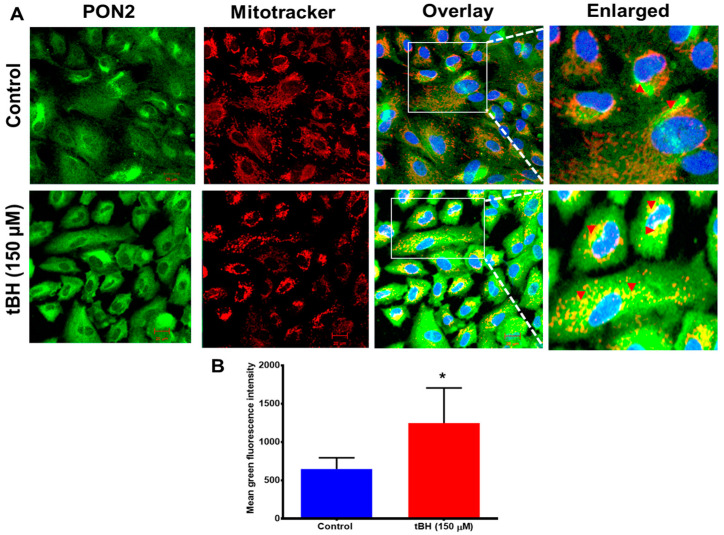
PON2 colocalizes with mitochondria in RPE cells. (**A**) Primary cultured human RPE cells were treated with tBH (150 µM) for 4 h and double-stained with PON2 (green) and mitochondrial tracker (red). Nuclei were stained with DAPI (blue). Colocalization is visualized by yellow staining in the overlayed and enlarged images for PON2 and mitochondria in RPE cells (arrowheads). Compared to untreated control cells, the colocalization in tBH-treated cells appeared to be more pronounced (mean ± SD, n = 8–10). (**B**) Fluorescence intensity for PON2 was measured using Zen 2011 software. Scale bar: 20 μm. * *p* < 0.05.

**Figure 2:**
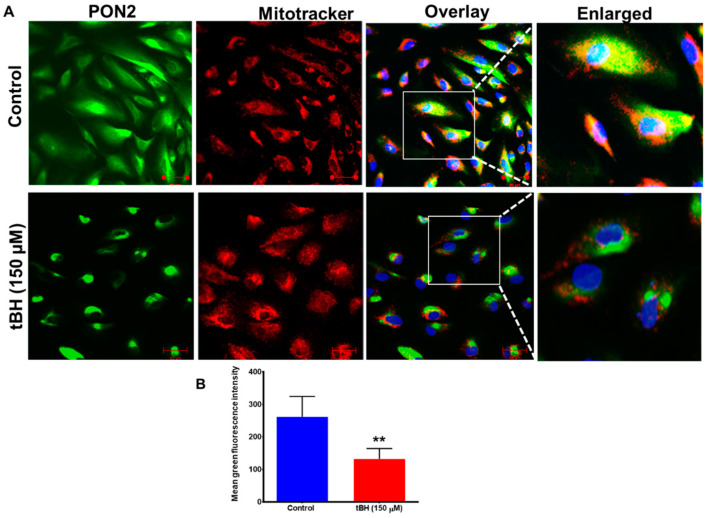
Decreased PON2 expression with 24 h treatment with oxidant tBH. (**A**) Primary cultured RPE cells were treated with tBH (150 μM) for 24 h and stained with PON2 and mitochondrial tracker. Nuclei were stained with DAPI (blue). Yellow staining in the composite and enlarged image for PON2 (green) and mitochondria (red) in RPE cells (arrows) highlights colocalization. The images on the right are enlarged to make it easier to see the pixels showing colocalization. (**B**) The green fluorescence intensity measured with ZEN 2010 showed a significant decrease in PON2 green intensity with tBH treatment (mean ± SD, n = 8–10). When compared to untreated control cells, the colocalization in tBH-treated cells seemed to be more pronounced. Scale bar: 50 μm. ** *p* < 0.01.

The PON2 protein is distributed throughout the cytoplasm in hRPE cells, and greater staining was seen perinuclearly, where it is colocalized with mitochondria (Figure 1 and Figure 2A). Oxidative stress from tBH (150 µM) treatment for 4 h caused a significant increase in the expression of PON2 along with a pronounced mitochondrial colocalization, as evident in the merged image (Figure 1A,B). Immunostaining results were confirmed by PON2 mRNA and protein expression from hRPE cells that had been exposed to tBH for 4 h; the results revealed a significantly higher level of PON2 expression at both the transcript and protein levels (Supplementary Figure S1A–C).

Expression of PON2 significantly decreased 24 h after tBH treatment, as seen by immunostaining (Figure 2A,B), qPCR and immunoblot analysis (Supplementary Figure S2A–C).

### 3.2. Mitochondrial Bioenergetics Is Altered in tBH Treated PON2 Knockdown RPE Cells

AMD and other age-related disorders have been reported to be linked to mitochondrial dysfunction. Therefore, assessing mitochondrial respiratory characteristics is a valuable approach to studying pathological states. The electron transport chain is the primary molecular pathway for generating electrons, protons, and ATP and consuming oxygen. Mito stress assays quantify mitochondrial basal oxygen consumption, ATP-linked respiration, maximal respiration, and spare respiratory capacity.

**Figure 3:**
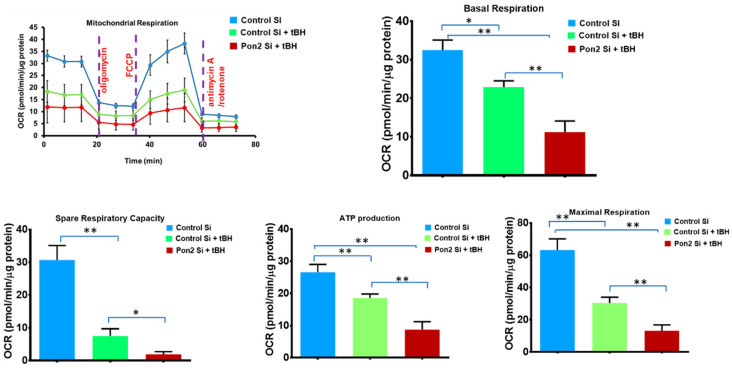
PON2 knockdown causes mitochondrial bioenergy deficiency in stressed RPE Cells. RPE cells from control and PON2-silenced groups were treated with tBH (300 µM) for 24 h. Under basal conditions and in response to the mitochondrial inhibitors oligomycin, cyanide-4-(trifluoromethoxy) phenylhydrazone, and antimycin A plus rotenone, and oxygen consumption rate was evaluated in real-time using the Seahorse XFe96 Flux Analyzer. The measurement of basal respiration, spare respiratory capacity, ATP-linked respiration (ATP-production), and maximal respiration is shown in bar graphs, n = 6–7 wells per treatment group, and values are expressed as means ± SEM, * *p* < 0.05, ** *p* < 0.01.

At first, we verified PON2 silencing by using qPCR. PON2 mRNA expression was reduced by 80% in the PON2-silenced RPE cells (Supplementary Figure S3). Functional analysis showed that tBH-treated RPE cells had mitochondrial dysfunction as indicated by impairments in baseline respiration, ATP-linked respiration, spare respiratory capacity, and a nearly 70% decrease in maximal respiration (Figure 3). In comparison to control cells stimulated with tBH, maximal respiration was significantly reduced in PON2-silenced cells. Similarly, PON2 knockdown cells treated with tBH had a cellular ATP concentration that was much reduced (90% lower). PON2 knockdown considerably impaired all bioenergetic metrics, suggesting a crucial function for PON2 in RPE cells. No significant difference was found between control Si and Pon2 silenced RPE cells in bioenergetic parameters such as basal respiration, ATP production, and maximal respiration (Supplementary Figure S4).

### 3.3. PON2-Deficiency Accelerates Retinal Degeneration Induced by NaIO_3_

**Figure 4:**
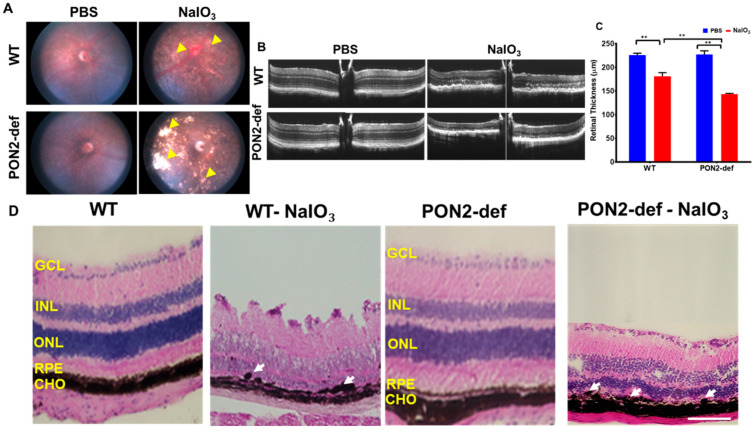
Changes in the retinal structure of WT and PON2-deficient mice after NaIO_3_ treatment. NaIO_3_ (20 mg/kg BW) was injected into 8-week-old mice via the tail vein, and on Day 7, the fundus (**A**), OCT (**B**,**C**), and histology (**D**) were performed. In all NaIO_3_-treated groups, retinal atrophy and thinning were observed. The retinal degeneration, however, was more severe in PON2-deficient mice as shown by larger white patches ((**A**); see yellow arrowheads). When NaIO_3_ was administered to WT mice, the retinal thickness was significantly reduced (**B**,**C**), and this effect was further decreased in PON2 -def mice (**B**,**C**). Histology of the retina demonstrated RPE degeneration (white arrows) and retinal thinning (**D**). The RPE monolayer was damaged in the NaIO_3_-treated groups, and the RPE cells showed a rounded, degenerative phenotype (see arrows). PON2-deficient mice, on the other hand, showed substantial RPE loss, distorted and thinned ONL, and disorganized INL following NaIO_3_ injection. Representative images from 5–6 mice are shown. Scale bar: 25 µm. (mean ± SD, n = 5–6; ** *p* < 0.01). GCL: Ganglion cell layer, INL: Inner nuclear layer, ONL: Outer nuclear layer, RPE: Retinal pigment epithelium, CHO: Choroid.

We used a murine model of NaIO_3_ which causes RPE to degenerate, identical to what is seen in atrophic AMD for these studies in vivo. We obtained color fundus photographs on day 7 to evaluate how the retina responded to NaIO_3_. NaIO_3_ administration was associated with retinal degeneration and significant changes in the RPE layer could be visible in the fundus images (yellow arrowheads). PON2-deficient mice displayed higher RPE degeneration-related lesions with NaIO_3_ than the corresponding WT mice (Figure 4A).

A spectral domain OCT imaging device was used to acquire an in vivo visualization of retinal structure. Representative OCT images are shown for mice in Figure 4B. Well-defined retinal layers are evident in the PBS-injected group (Figure 4B). By day 7 post-NaIO_3_ administration, OCT images show severe outer retinal layer damage in addition to a blurring of the layer boundaries. The NaIO_3_-treated PON2-deficient mice had significantly thinner retinas (Figure 4C).

We processed the posterior eye cups for histology to analyze the structural changes in the retina that occurred after post-NaIO_3_ injection (Figure 4D). In mice administered with PBS, no apparent structural alterations were observed. PON2-deficient mice challenged with NaIO_3_ had RPE cells that were bigger, more rounded, vacuolized, and causing separation from the entire RPE monolayer (Figure 4D, arrows). In all NaIO_3_-treated groups, ONL and INL had total distortion, and as seen in the representative pictures, PON2-deficient mice incurred more severe damage, with nearly entire degeneration of the cone and rod photoreceptor cells. Most of the photoreceptors and their outer segments were destroyed, coinciding with the findings of visual functional loss by ERG shown in the next figure.

### 3.4. PON2-Deficient Mice Display Altered Scotopic and Photopic ERGs

**Figure 5:**
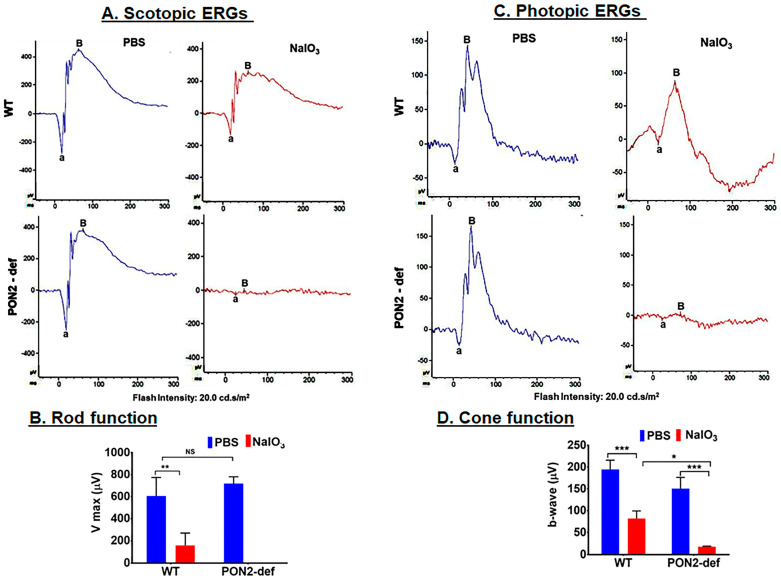
Changes in scotopic and photopic ‘a and b’ wave amplitudes following 20 mg/kg of NaIO_3_ administration in WT and PON2-deficient mice. Amplitudes of scotopic a- and b-waves were significantly reduced 7 days after injection with NaIO_3_ by more than two-fold in WT mice and a complete suppression of scotopic ERG in PON2-deficient mice (**A**,**B**). The amplitudes of photopic ERGs were also significantly compromised with NaIO_3_ treatment; however, the reduction in amplitude was more severe in PON2-deficient mice (**C**,**D**). The results are presented as the mean ± (SD). n = 3 mice. NS: Not significant. * *p* < 0.05, ** *p* < 0.01 *** *p* < 0.001.

Representative ERG recordings to a relatively bright flash (0.02 cd-s/m^2^) are shown for scotopic (Figure 5A,B) and photopic conditions (Figure 5C,D). Considering first the dark-adapted ERG (Figure 5A), the ERG was robust and well-defined for WT control and PON2-deficient mice. When analyzing Vmax for these groups of mice, even though there was an increase in the PON2-deficient mice, this increase was not significant. When treated with NaIO_3_, the ERG was essentially non-detectable for the PON2-deficient mice, but clearly recordable with well-defined features, for the WT mice. While the difference between Vmax before and after treatment with NaIO_3_ was significant for both strains, the decrease in retinal function was severely compromised in PON2-deficient mice when compared to corresponding WT (Figure 5B). A similar pattern of results was found when examining cone-mediated function (Figure 5C,D). These findings imply a protective role for PON2 in both rod- and cone-mediated retinal function.

### 3.5. PON2-Deficient Exacerbates Cell Death in Mouse Retina

**Figure 6:**
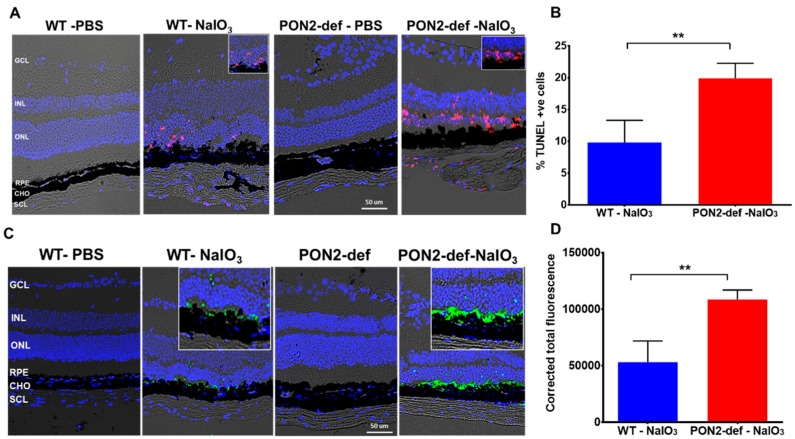
PON2-deficient retina is more vulnerable to NaIO_3_ injury-induced cell death. Seven days after receiving a single dose of NaIO_3_ (20 mg/kg), TUNEL and caspase activation were observed in the retina of WT and PON2-deficient mice. (**A**) Apoptotic cells (pink) are observed mostly in the ONL (see also insets). No cell death was observed in PBS-treated WT or PON2-deficient mice. In the PON2-deficient + NaIO_3_ group, the percentage of TUNEL-positive cells was significantly greater (**B**). Data are shown as mean ± SEM (n = 9, ** *p* < 0.001). (**C**) Activation of caspase-3 in NaIO_3_-challenged mouse retina (see also insets). There was no indication of an activated caspase-3 signal in the PBS-treated groups. (**D**) Quantification revealed that caspase-3 activation was significantly higher in NaIO_3_-treated PON2-deficient mice when compared with the comparable stressed WT mice (mean ± SEM, n = 9, ** *p* < 0.001). GCL: Ganglion cell layer, INL: Inner nuclear layer, ONL: Outer nuclear layer, RPE: Retinal pigment epithelium, CHO: Choroid, SCL: Sclera.

Retinal functional and imaging studies demonstrated significantly increased retinal thinning and dysregulated retinal function in the PON2-deficient mice challenged with NaIO_3_. To investigate cell death, we used TUNEL labeling, which detects DNA fragmentation during apoptosis. TUNEL-positive cells (red) were not observed in the PBS-treated sections (Figure 6A) but were considerably increased after exposure to NaIO_3_. TUNEL-positive cells were mostly seen in the outer nuclear layer (ONL), where the photoreceptor nuclei reside. Quantification of the TUNEL-positive cells revealed 10% cell death in WT mice given NaIO_3_, whereas the percentage doubled in PON2-deficient mice, showing that degeneration occurs more quickly in the absence of PON2. Next, we used active caspase-3 staining to examine the nature of cell death in this model. There was no evidence of active caspase-3 expression in the PBS-treated sections. On day 7 after NaIO_3_ treatment, cleaved caspase-3-positive cells (green staining in Figure 6C,D) were seen in the RPE layer along with a few positive cells in the ONL of WT mice. However, cleaved caspase-3 expression was higher in the NaIO_3_-treated PON2-deficient mice, and most of the degenerated, rounded RPE cells were stained positive. A greater number of ONL cells are also stained positive for active caspase-3 (Figure 6B). When compared to comparable WT, the fluorescence intensity for cleaved caspase-3 was significantly higher in PON2-deficient mice treated with NaIO_3_ (Figure 6D). Importantly, we found that PON2-deficient mice have increased active caspase-3 expression, which could help explain why they are more susceptible to apoptosis with stress.

## 4. Discussion

PON2 is a multipotent enzyme with both enzymatic and non-enzymatic actions that have been linked to the pathology of several disorders. PON2 plays an important role in the regulation of oxidative stress, which is a key factor in the pathophysiology of AMD. In this study, we present the first evidence showing that (a) in hRPE cells, PON2 expression and localization are regulated by oxidative stress; (b) PON2 deficient hRPE cells have significantly dysregulated mitochondrial activity; (c) PON2-deficient mice are more vulnerable to NaIO_3_-induced retinal pathology as shown by alteration in retinal structure and function; and d) cell death was accelerated in NaIO_3_-injected PON2-deficient mice.

Phylogenetic analysis suggests that PON2 is the oldest member of the family and is a ubiquitously expressed intracellular enzyme [7]. PON2 is expressed in a variety of tissues, including the liver, lung, kidney, heart, pancreas, small intestine, muscle, testis, endothelial cells, tracheal epithelial cells, macrophages, retina, and brain [7,26,27,28,29,30,31]. Sub-cellular distribution studies have shown that PON2 is localized primarily in the mitochondria and the endoplasmic reticulum [9,16]. In the primary RPE cells, PON2 is mainly associated with mitochondria, cytosol and some in the nuclei. We also show that oxidative stress has an impact on the regulation of PON2 in hRPE cells, a similar finding was reported in different cell types [16,31,32].

PON2 polymorphism has been linked to a variety of illnesses [33]. Ala/Gly at position 148 and Ser/Cys at position 311 are two of the polymorphisms of PON2 that are often detected [26] and these polymorphisms are associated with its decreased lactonase activity [34]. It appears likely that the existence of mutations at the 148 and 311 sites could be regarded as a risk factor for the onset of cataracts [35]. The PON2 gene polymorphisms A148G and S311C have been independently linked to diabetic nephropathy in type II diabetic patients [36]. PON2 Cys311Ser polymorphism is also linked with Alzheimer’s disease. PON2 311Ser, along with another gene, the apoE4 allele, helps in the development of AD and vascular dementia [37,38]. Whether these two polymorphisms play a role in the development or progression of AMD needs detailed investigations.

Given the growing importance and function of PON2 in free radical defense, we anticipated a significant regulation of PON2 status after oxidative stress exposure. The initial increase in PON2 at 4 h after tBH treatment and its enhanced expression in mitochondria in the present study could represent compensatory mechanisms to better protect cells from injury caused by stressors. At 24 h after tBH treatment, PON2 expression in RPE cells was found to decrease (Figure 2). A decrease in PON2 expression at the transcript and protein level and decreased lactonase activity were observed in high-glucose-treated Caco-2 cells [32] and in advanced glycation end products exposed to endothelial cells [12]. Human retinal endothelial cells and ARPE-19 cells both displayed decreased PON2 protein levels after receiving N-(carboxymethyl) lysine (CML) treatment [13]. In human diabetic retinas, similar decreased PON2 levels were found [13]. Moreover, a lower PON2 expression has been observed in the liver and heart of hyperglycemic animal models [39,40]. The antiapoptotic activity of PON2 is cell-type-specific and involves many signaling pathways. For instance, the JNK-Fis1 signaling pathway is activated in ARPE-19 and endothelial cells [13], and the PI3K/Akt/GSK-3 RISK pathway is involved in cardiomyocytes [41]. PON2 deficiency increases the expression of Fis1 resulting in the fragmentation of mitochondria and increases the production of ROS and activates the release of Cyt-c [13]. Furthermore, it has been reported that PON2 deficiency resulted in the downregulation of mitophagy markers such as PARKIN, PINK1, and BNIP3 as well as the mitophagy-specific cargo receptors PRKCD and DNM1 [42]. As previously mentioned, moderate to extremely high reactant concentrations decrease the activities of antioxidants because they impair the molecular machinery required to activate these enzymes [43]. Our present studies reveal that PON2 expression was considerably upregulated in mitochondria, and this predominant location is consistent with its function in mitoprotection, most likely through scavenging reactive oxygen species. However, detailed mechanistic studies on its protective mechanism(s) involving RPE need to be undertaken.

It is well known that mitochondria play an active part in lipid and glucose metabolism, and are significant generators of ROS, which are typically eliminated by endogenous antioxidants present in the mitochondria [44]. PON2 has been shown to modulate mitochondrial function and exert antiapoptotic properties [13,16,45,46]. PON2 and cardiolipin are located in the inner mitochondrial membrane, and in the absence of PON2, oxidative stress leads to cardiolipin peroxidation, which causes mitochondrial dysfunction [11]. Our results provide strong evidence for the notion that PON2 deficiency severely impaired ATP synthesis as well as other respiratory metrics such as maximal respiration and spare respiratory capacity. White adipose tissue [47] and peritoneal macrophages [16] obtained from PON2-deficient mice both showed similar decreased mitochondrial respiration and ATP generation. PON2 deficiency alters mitochondrial oxidative stress by increasing mitochondrial superoxide production, increasing lipid peroxidation, and decreasing reduced glutathione levels [16]. It also alters mitochondrial function by decreasing complex I and III activity and total ATP levels. Additionally, it has been noted that overexpressing PON2 protects cells from apoptosis, improves mitochondrial activity, and reduces cardiolipin peroxidation [11,13,16].

While reports exist to show that PON2 is a key regulator of oxidative stress in non-ocular tissues, the effects of PON2 deficiency in the retina have not garnered much attention. The current work is the first to explore the effects of PON2 deficiency in the retina and how, in the absence of it, oxidative stress affects retinal structure and function. NaIO_3_ was administered to mice to test the impact of PON2 deficiency on the retina. There is currently no single rodent animal model that replicates all the hallmark signs of AMD in a progressive manner. Due to its RPE-specific toxicity and the RPE changes observed during AMD progression, the NaIO_3_ model is the most widely used preclinical mouse model of dry AMD (non-exudative) [21,25,48,49,50,51]. RPE loss from exposure to NaIO_3_ in the retina results in secondary damage to the photoreceptors, which is analogous to how RPE dysfunction in patients with dry AMD causes photoreceptor cell death [52]. This model has been successfully used on a variety of mammalian species, including sheep [53], pigs [54], rabbits [55], and monkeys [56]. We used systemic administration of NaIO_3_, which has been shown to be a successful way to induce retinal degeneration in mouse models [21,25,48,57,58,59,60,61].

Our studies revealed that PON2 deficiency alone has no effect on retinal structure or function. The PON2-deficient mouse is not a full knockout model and the tissue expression of PON2 is roughly 5–10% that of wild-type mice [22]. It is unknown how much PON2 must be lost for a system to become dysfunctional. The deficient model does, however, have translational advantages since PON2 polymorphisms in the general population are thought to decrease PON2′s enzymatic activity [62]. Our imaging and retinal histology findings demonstrate that PON2-deficient mice are extremely susceptible to retinal damage induced by oxidative stress. In comparison to corresponding WT mice, fundus imaging revealed bigger lesions, OCT revealed retinal thinning, and histology revealed greater patchy RPE cell loss as well as disorganization and photoreceptor deterioration. The scotopic a- and b-wave responses in PON2- deficient mice appeared to plateau, in contrast to the considerably reduced scotopic a- and b-wave responses in WT treated with NaIO_3_, which provided additional support for the imaging results. Rod photoreceptors may be more susceptible to damage from oxidative stress than cones because of changes in fatty acid compositions, metabolic needs, and bioenergetic needs [63,64]. Similar results have been found in animals lacking the Nrf2 gene that had diminished rod function [65]. The photopic a- and b-waves are severely hampered in WT, and this hampered state was further exacerbated in PON2-deficient mice exposed to NaIO_3_. In diabetic mice, rod a- and b-wave ERGs were reduced by half, although cone a- and b-wave ERGs remained unchanged, demonstrating the differential susceptibility of rods and cones to oxidative stressors [66]. It is intriguing to note that a recent study found that PON2-deficient mice showed poor motor coordination and notable biochemical changes at the mRNA level, which may influence several cellular activities in the brain [67]. However, additional omics studies are ongoing to dissect the key pathways regulated under baseline and stressed conditions.

In the current study, we demonstrated that compared to WT mice with retinal degeneration, PON2-deficient mice had significantly more TUNEL-positive cells and activated caspase-3. Previous studies have demonstrated that apoptosis plays an important role in RPE degeneration in NaIO_3_-challenged mice [21,25]. Additionally, we demonstrated that NaIO_3_ treatment significantly reduces RPE transepithelial resistance, which is linked to a disruption of tight junction proteins. PON2 is in the inner mitochondrial membrane, where it controls the activity of the respiratory complex and lowers the amounts of superoxide in the mitochondria [16]. Our in vitro tests corroborate the observations, which show that PON2-knockdown RPE cells have significantly reduced mitochondrial function. Similar findings of increased oxidative stress susceptibility with PON2 deficiency have been reported in numerous disease models, including atherosclerosis [16,22], heart failure [68], impaired hepatic insulin signaling [69], and obesity [47]. The protective effects of PON2 in different animals are partially mediated by its role in mitochondrial function [16]. It will be of interest to determine the role of PON2 in the regulation of autophagy/mitophagy under stressed and pathological conditions. One other area worthy of investigation will be to study the regulatory role of PON2 in lipid peroxidation in the RPE/retina. Previous work in non-ocular tissues showed that overexpression of PON2 inhibited cell-mediated low-density lipoprotein oxidation [15]. Work along these lines is ongoing in our laboratories.

## 5. Conclusions

**Figure 7:**
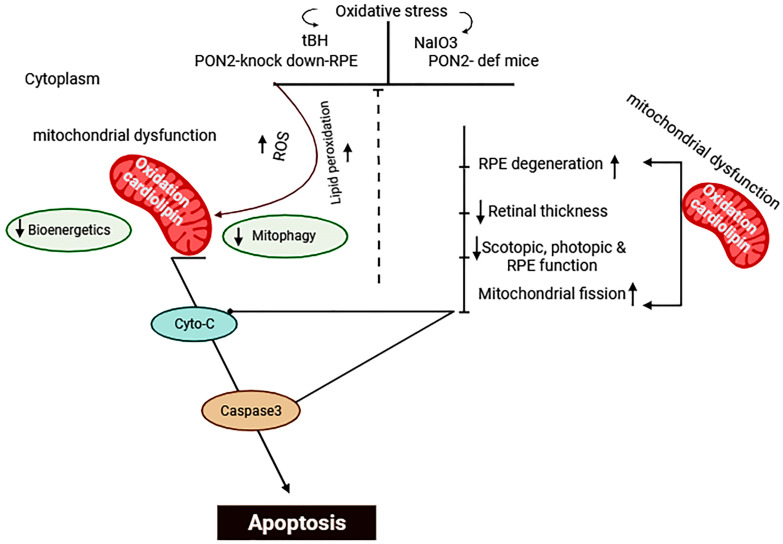
Scheme illustrating regulation of apoptosis and mitochondrial function by PON2 in the RPE/retina. Created with BioRender.com.

In summary, our work showed that oxidative stress regulates PON2 in a time-dependent manner. As depicted in the scheme shown in Figure 7, oxidative stress increased mitochondrial PON2, and silencing PON2 in RPE cells significantly compromised the bioenergetic capabilities of the mitochondria. The markedly impaired retinal function, structure, and increased apoptosis in PON2-deficient mice demonstrate their extreme susceptibility to NaIO3-induced stress. Taken together, these data provide the first in vivo proof of evidence of the role of PON2 in a model of dry AMD. However, a deeper understanding of the signaling pathways of PON2 action needs to be pursued, and ways to elevate PON2 levels as a possible treatment for AMD or other retinal diseases may prove to be a valuable approach.

## Supplementary Materials

The following supporting information can be downloaded at: https://www.mdpi.com/article/10.3390/antiox12101820/s1, Figure S1: PON2 protein expression in hRPE cells exposed to tBH for 4 h, Figure S2: PON2 expression in hRPE cells exposed to tBH for 24 h, Figure S3: PON2 mRNA expression. Figure S4: The mitochondrial bioenergetics in Pon2 silenced RPE cells.
