# Supplementary material for: Paraoxonase 2 Deficiency Causes Mitochondrial Dysfunction in Retinal Pigment Epithelial Cells and Retinal Degeneration in Mice
Source: Antioxidants (Basel). 2023 Sep 30;12(10):1820. doi: 10.3390/antiox12101820 (PMC10604559; doi:10.3390/antiox12101820)
Supplement: Supplementary file 1 [file antioxidants-12-01820-s001.zip › antioxidants-2592654-supplementary.pdf]

antioxidants

Supplementary Method

Immunoblot Analysis

RPE cells grown to confluence on 6-well culture plates (VWR) were treated with tBH (150 µM) for 4 h or 24 h. Protein was extracted from the cells using mammalian protein extraction reagent (# 78501, Thermo Scientific) with Halt Protease and Phosphatase Inhibitor Cocktail (#78442, ThermoFisher Scientific) and quantified with a protein assay (Bio-Rad, Hercules, CA). Protein was resolved in equal amounts on 10% mini-PROTEAN TGX gels (# 4561034, BioRad) and then transferred to PVDF blotting membranes. Membranes were probed overnight at 4°C with rabbit polyclonal anti-PON2 antibody (#LSC163863, LS Bio, Shirley, MA). Signals were detected using an ECL chemiluminescence system (ThermoFisher) following incubation with the corresponding secondary antibody tagged with horseradish peroxidase. Membranes were then stripped and reprobed with mouse monoclonal anti-GAPDH (Millipore, Billerica, MA). Image J was used to measure the protein band intensity, which was then normalized using the loading control.

Supplementary Figure S1

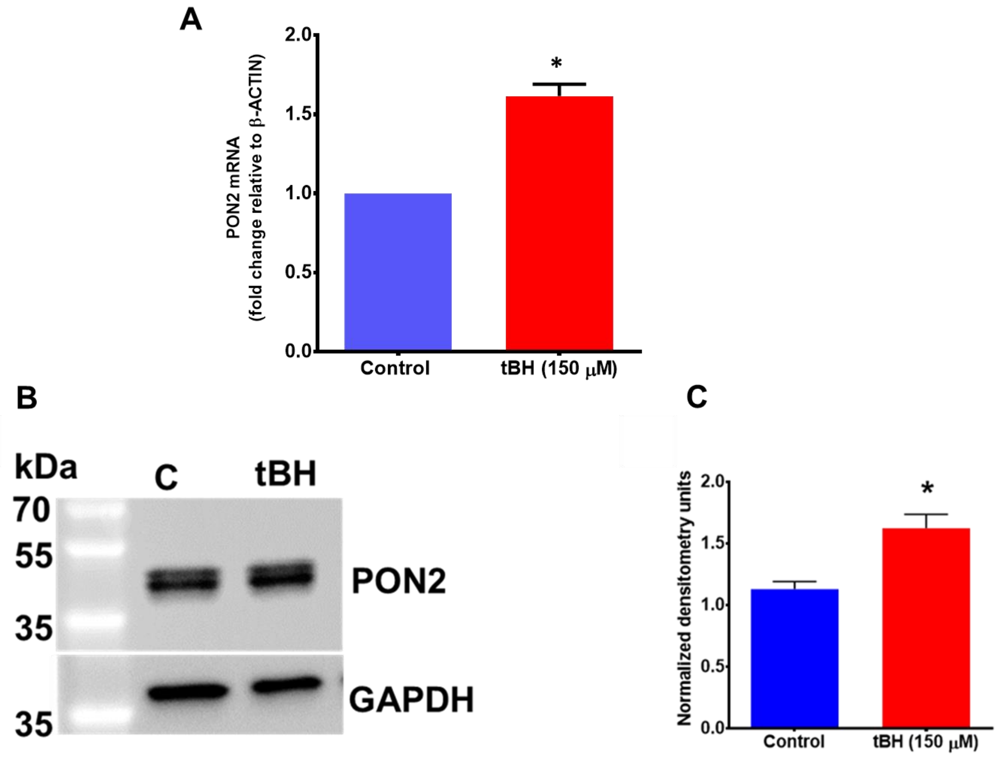

Supplementary Figure S1. PON2 mRNA and protein expression in hRPE cells exposed to tBH for 4h.

A. PON2 mRNA expression in RPE cells treated with tBH (150µM) for 4 h Total RNA was isolated 4 h after tBH treatment and qRT-PCR analysis was performed to detect PON2 fold change relative to ß-ACTIN levels. **B**. Protein extracted from RPE cells treated with tBH (150 µM) for 4 h was subjected to immunoblot analysis and probed for PON2 expression. C. Densitometric analysis of the bands showed a significant increase in PON2 expression with tBH vs. control (n = 3, Mean ± SEM), * $P<0.05$).

Supplementary Figure S2.

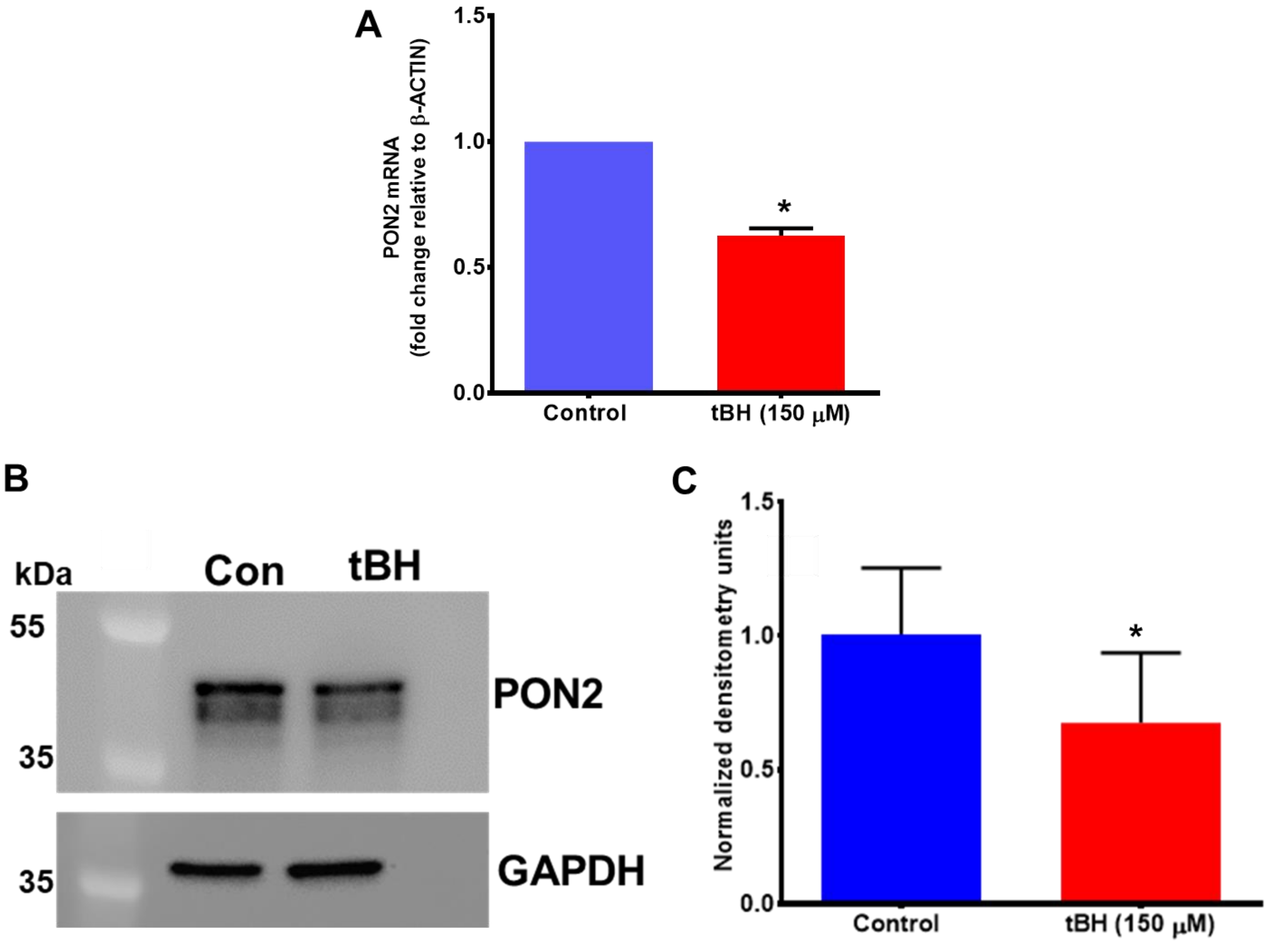

Supplementary Figure S2. PON2 mRNA and protein expression in hRPE cells exposed to tBH for 24h. A. PON2 mRNA expression in RPE cells treated with tBH (150μM) for 24 h Total RNA was isolated 24 h after tBH and qRT-PCR analysis was performed to detect PON2 fold change relative to ß-ACTIN levels (n=3, Mean ±SEM). A. Western blot analysis of RPE cells under tBH stress (24 h) showed a significant reduction in PON2 expression. **B**. Densitometric analysis of the bands showed a significant decrease in PON2 expression over control (n=4, data mean ± SEM) * P<0.05.

Supplementary Figure S3

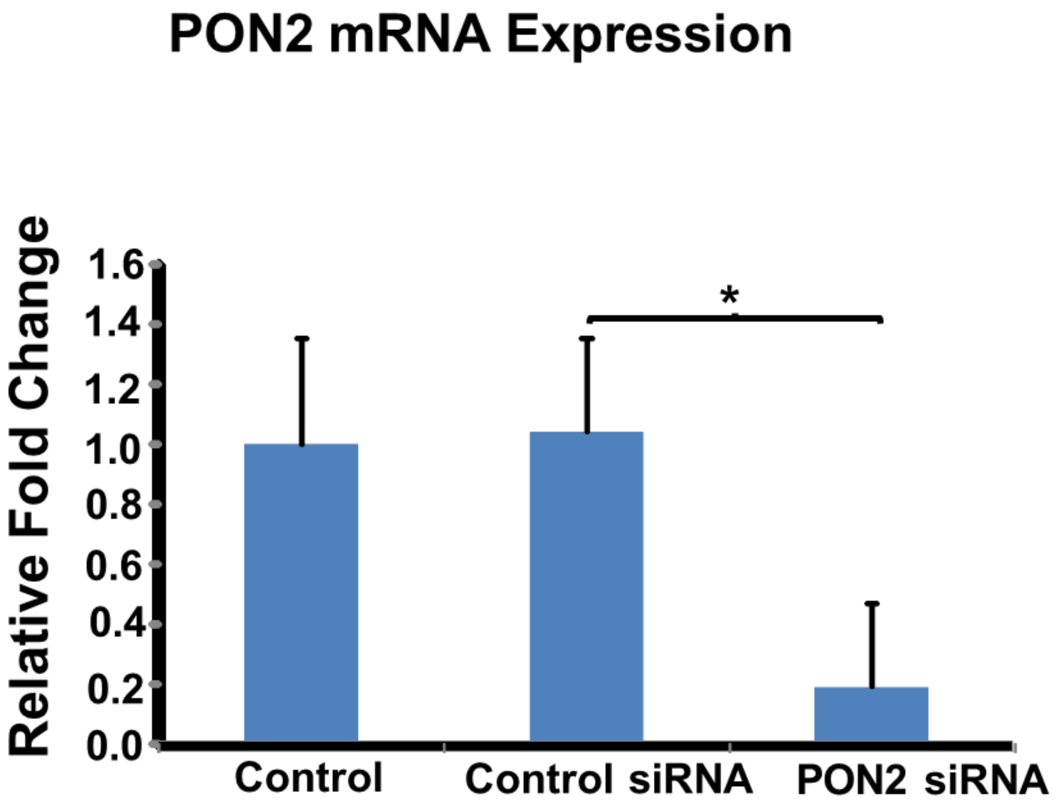

Supplementary Figure S3. Validation of silencing of PON2 in ARPE19 cells. RPE cells were transfected with negative control siRNA (10 nM) or PON2 siRNA (10 nM). The expression of PON2 mRNA in relation to GAPDH 24 h following transfection is shown (mean ± SEM, n = 3, ** $p < 0.01$).

Supplementary Figure S4

The mitochondrial bioenergetics in Pon2 silenced RPE cells

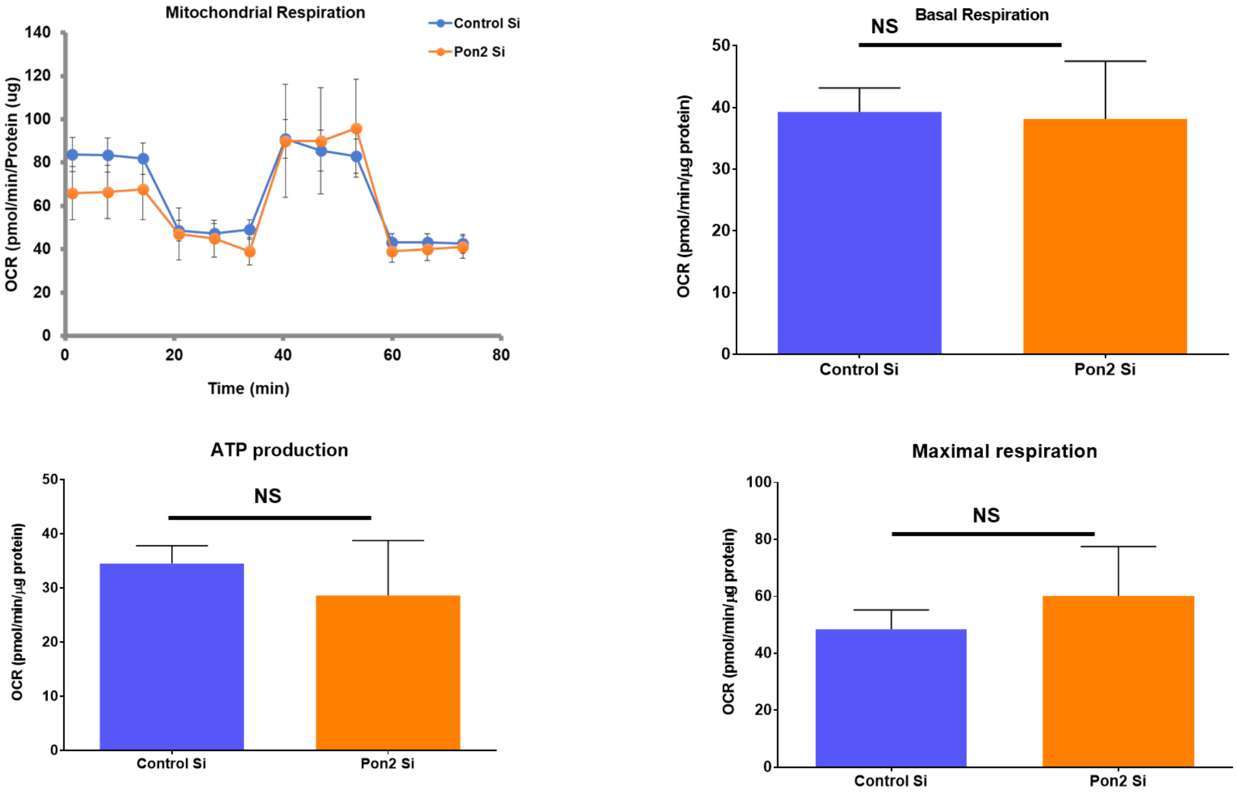

Supplementary Figure S4. PON2 silencing alone does not alter mitochondrial function in RPE Cells. Analysis of mitochondrial bioenergetics was done on RPE cells from control Si and PON2-silenced groups. Under basal conditions and in response to the mitochondrial inhibitors oligomycin, cyanide-4-(trifluoromethoxy) phenylhydrazone, and antimycin A plus rotenone, Oxygen consumption rate was evaluated in real-time using the Seahorse XFe96 Flux Analyzer. The measurement of basal respiration, ATP-linked respiration (ATP-production), and maximal respiration is shown in bar graphs, N = 4-5 wells per group, and values are expressed as means ± SEM. NS: Not Significant.
